# Bolus characteristics based on Magnetic Resonance Angiography

**DOI:** 10.1186/1475-925X-5-53

**Published:** 2006-10-17

**Authors:** Zhijun Cai, Alan Stolpen, Melhem J Sharafuddin, Robert McCabe, Henri Bai, Tom Potts, Michael Vannier, Debiao Li, Xiaoming Bi, James Bennett, Jafar Golzarian, Shiliang Sun, Ge Wang, Er-Wei Bai

**Affiliations:** 1 Department of Electrical and Computer Engineering, University of Iowa, Iowa City, IA, USA; 2 Department of Radiology, University of Iowa, Iowa City, IA, USA; 3 University of Iowa Hospital and Clinic, Iowa City, IA, USA; 4 Department of Computer Engineering, Iowa State University, Ames, IA, USA; 5 Department of Radiology, University of Chicago, Chicago, IL, USA; 6 Department of Radiology and Biomedical Engineering Northwestern University, Chicago, IL, USA

## Abstract

**Background:**

A detailed contrast bolus propagation model is essential for optimizing bolus-chasing Computed Tomography Angiography (CTA). Bolus characteristics were studied using bolus-timing datasets from Magnetic Resonance Angiography (MRA) for adaptive controller design and validation.

**Methods:**

MRA bolus-timing datasets of the aorta in thirty patients were analyzed by a program developed with MATLAB. Bolus characteristics, such as peak position, dispersion and bolus velocity, were studied. The bolus profile was fit to a convolution function, which would serve as a mathematical model of bolus propagation in future controller design.

**Results:**

The maximum speed of the bolus in the aorta ranged from 5–13 cm/s and the dwell time ranged from 7–13 seconds. Bolus characteristics were well described by the proposed propagation model, which included the exact functional relationships between the parameters and aortic location.

**Conclusion:**

The convolution function describes bolus dynamics reasonably well and could be used to implement the adaptive controller design.

## Background

To diagnose vascular disease, high quality imaging is extremely important. Many imaging studies utilized an intravascular injection of contrast material that must be delivered to the field-of-view (FOV). For a typical four channel CT scanner, the FOV is the imaging aperture, which has a width of about 10 mm [1]. Estimating the peak density of the contrast bolus during the entire period of data acquisition is highly desirable for planning image acquisition, processing, and display. A detailed knowledge of bolus characteristics helps to optimize vascular imaging and thereby improve depiction of vessels, lesions, and tumors [2-5].

Currently, Magnetic Resonance Imaging (MRI), digital subtraction fluoroscopy and CT are three imaging modalities used for angiography. One determinant of image quality is the accurate spatial and temporal synchronization of the peak bolus density with the imaging acquisition window. For CT, this involves translating the scanner table appropriately after intravascular injection [6,7]. MRA and Digital Subtraction Angiography (DSA) have a relatively large FOV, which makes the synchronization easier. However, compared to CTA, MRA has inferior spatial resolution. DSA has the best spatial resolution, but it is invasive, and 2D projection images may limit its diagnostic utility. Although many researchers have investigated bolus chasing in DSA [8] and MRA [9,10], there has been insufficient work devoted to synchronizing contrast material delivery and propagation with the CT imaging aperture. Our group has previously proposed the adaptive bolus chasing control scheme to address this issue [11].

The goal of this paper is to investigate arterial bolus characteristics (focusing on the aorta) in order to develop an adaptive control mechanism for bolus-chasing CTA. Properties of the contrast bolus, such as bolus velocity, peak position, and dispersion, were investigated. Using sequential images from MRA contrast bolus timing datasets, temporal density curves and spatial density curves were extracted. These curves were displayed as a bolus Distance-Time-Density profile and used to formulate a mathematical model.

As it travels through the artery, the bolus fills the vessel for some length. Therefore, a complete description of bolus dynamics requires a large field of view. CT images a cross-section of the body, usually in the transverse plane, and thus provides information about the bolus density in only a single slice at any given time. CT is incapable of interrogating a bolus along the length of an artery that travels orthogonal to the imaging plane. Because they offer a large FOV, both MRA and DSA could potentially provide this information. Of these, MRA is our first choice due to similarities with CTA regarding the contrast injection site, and the likelihood that both have similar bolus dynamics. In addition, MRA does not expose human subjects to ionizing radiation or nephrotoxic contrast agents.

Quantitative analysis of MRA data is difficult because of the complex relationship between MR signal intensity and contrast material concentration or density. However, the analysis can be simplified by using relative (as opposed to absolute) contrast bolus density, wherein a linear relationship between signal intensity and contrast density is assumed. Other assumptions used in our work are: 1) the MRA imaging plane is assumed to pass through the middle of the aorta, thereby neglecting effects due to vessel curvature; 2) the effects of blood flow-related enhancement on MR signal intensity are not considered. Using the assumptions above, the pixel value (after subtraction of background) in the MRA image corresponds to the relative bolus density. Through this paper, "density" refers to the relative bolus density.

In this paper, the procedure for analyzing the MRA images will be described first, followed by an analysis of the bolus characteristics. A complete description of the bolus dynamics is formulated by fitting the data to a set of standard mathematical functions; the results are then used to facilitate the development of an adaptive controller design.

## Methods

### Data acquisition and extraction

MRA datasets were collected from two sources: 1) Routine clinical MRA studies with bolus timing runs performed on patients at the University of Iowa Hospital and Clinics (UIHC), and 2) studies performed on volunteers at Northwestern University (NU). UIHC studies were performed on a 1.5 Tesla GE CV/i scanner using a 2D gradient echo sequence (TR = 5.7 ms, TE = 1.5 ms, 40 deg flip angle), 5 mm slice thickness, 38 cm FOV, 32 kHz bandwidth, and a 256 × 224 matrix. For these studies, a 3 cc contrast bolus (1.5 mmole gadodiamide) was injected into a peripheral vein followed by a 25 cc saline flush, both at a rate of 3 cc/sec. Northwestern University studies were performed on a 3 Tesla SIEMENS MR scanner using a 2D gradient echo sequence (TR = 4.2 ms, TE = 1.11 ms, 8 deg flip angle), 11 mm slice thickness, 40 cm FOV, and a 580 Hz/pixel bandwidth. A 2 cc contrast bolus was injected into a peripheral vein followed by a 10 cc saline flush, both at a rate 2 of cc/sec. At both sites, images were acquired in a sagittal or oblique sagittal plane through a long segment of the thoracoabdominal aorta. The header information in the Digital Imaging and Communications in Medicine (DICOM) format provided information about pixel spacing. All datasets were comprised of a series of image frames at one second time intervals. Patient information was also collected from DICOM, including gender and age.

From the large pool of bolus timing studies, we selected those demonstrating a clear, continuous longitudinal segment of the aorta. The bolus analysis algorithm was developed using MATLAB (ver. 7.1. [R14], SP3) with Image Processing Toolbox (ver. 5.1).

### Data analysis

**Figure 1:**
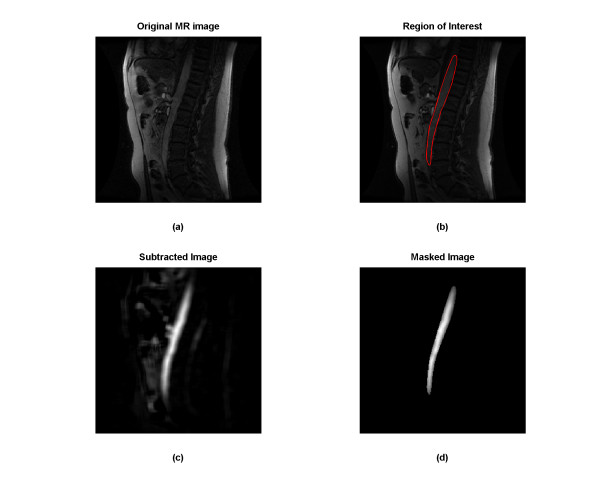
(a) The 18th frame from an original MRA bolus timing run collected at UIHC; (b) region of interest (ROI) for bolus; (c) subtracted image of (a) from the background; and (d) masked image for (c) (black pixel value is zero).

MR bolus timing runs were analyzed using the following procedure:

1) A region of interest **(ROI) **was placed on the frame that best depicted an extended longitudinal segment of the aorta (Figure 1a and 1b). Based on the above assumptions, we would set this **ROI **to all the frames;

2) Each frame was filtered with a 9 × 9 average (low pass) filter to reduce the noise;

3) Five to ten consecutive frames were averaged starting with the 3^rd ^frame and ending before the upslope of the contrast bolus. This average was used as the background and subtracted from each frame (Figure 1c);

4) The **ROI **from each frame was masked, that is, all the pixels outside the ROI was set to zero, while pixels inside the **ROI **untouched. (Figure 1d);

5) All pixel values in each row were summed and divided by the number of pixels in that row of the **ROI**. The result, for each frame, was a curve showing bolus average density of that cross section *versus *longitudinal displacement;

6) Steps 3–6, above, were repeated for each frame.

**Figure 2:**
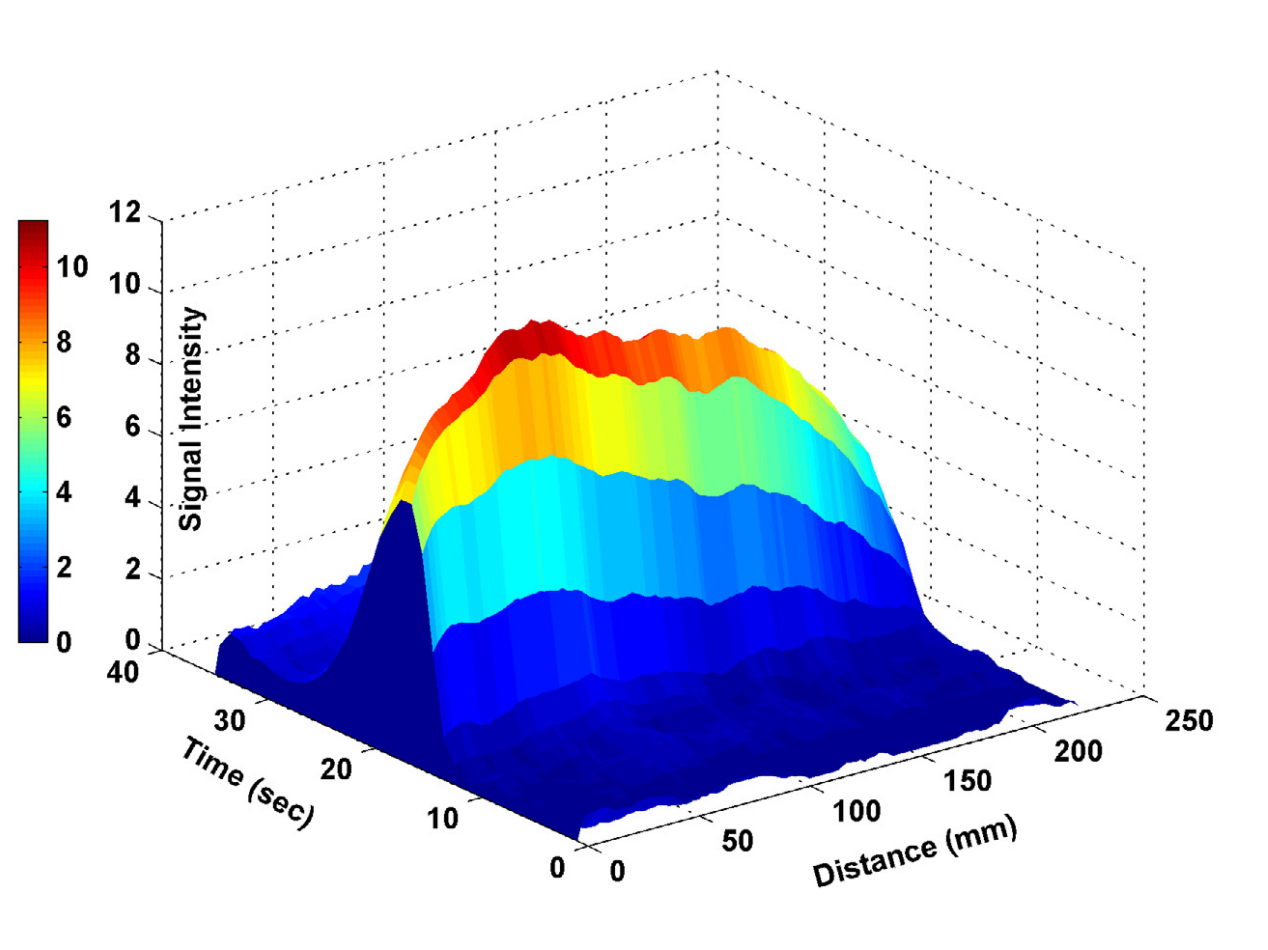
Bolus Distance-Time-Density profile in the ROI, where x-axis denotes the distance (zero is proximal row of the ROI), y-axis is time (zero is the antecubital vein injection time), and z-axis represents MR signal intensity.

The above procedure resulted in a Distance-Time-Density (3D) bolus profile, as shown in Figure 2, where the *x *axis denotes the longitudinal displacement along the aorta starting at its most proximal aspect in the ROI, the *y *axis denotes the time after the injection of the contrast material, and the *z *axis denotes the MR signal intensity (pixel value). With this information, the CT table movement requirement can be obtained. For example, in Figure 2, the contrast bolus remained in the aorta for a brief time, during which it traveled about 22 cm. A CT scanner table would need to cover the same distance in the same time period. This approach was used to facilitate and validate our adaptive controller design.

**Figure 3:**
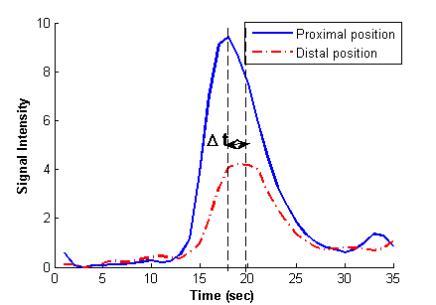
Bolus time density curve at proximal (solid) and distal (dash dot) positions of aorta in ROI. It is used to compute the peak-to-peak transit time *Δt*.

After obtaining the bolus Distance-Time-Density profile, the next step was to analyze the bolus characteristics. Although the bolus is conveyed by the human blood, its characteristics could be different from that of the blood because of its heavier density and dispersion property. Therefore, the reported blood velocity profile can not represent the bolus characteristics. And in general, bolus characteristics vary considerably among patients. To that end, the adaptive bolus chasing controller must be highly robust. The velocity profile plays an important role in the controller design. In this paper, bolus velocity was obtained by V=ΔzΔt
 MathType@MTEF@5@5@+=feaafiart1ev1aaatCvAUfKttLearuWrP9MDH5MBPbIqV92AaeXatLxBI9gBaebbnrfifHhDYfgasaacH8akY=wiFfYdH8Gipec8Eeeu0xXdbba9frFj0=OqFfea0dXdd9vqai=hGuQ8kuc9pgc9s8qqaq=dirpe0xb9q8qiLsFr0=vr0=vr0dc8meaabaqaciaacaGaaeqabaqabeGadaaakeaacqWGwbGvcqGH9aqpdaWcaaqaaiabfs5aejabdQha6bqaaiabfs5aejabdsha0baaaaa@34B1@, where Δ*z *is the distance between two selected positions inside the aorta and Δ*t *is the peak-to-peak transit time [12] (see Figure 3) of corresponding positions. The bolus we studied was very close to the heart, it flushed into the aorta very quickly (perhaps due to the one second frame rate). A greater Δ*z *may reduce the effect of noise. Therefore, the two positions (selected for velocity computation) were chosen as the most proximal and distal of the ROI, respectively. In that sense, the velocity values obtained were more like averages. We also gave the "travel time" and "travel length" for each dataset. The former is defined as the dwell time of the bolus in the aorta for signal intensity greater than half-maximum. The latter denotes the longitudinal length of ROI that was analyzed.

### Data fitting

Our purpose was to study bolus characteristics by establishing a bolus propagation model that can be used to develop bolus-chasing CTA. Once an actual bolus 3D profile was obtained, the next step was to fit it into a mathematical model. Others have previously used a variety of functions to fit the bolus profile in order to find a bolus time-density curve at a specified location (i.e., temporal curve). For example, in [13], A gamma variate curve fitting C=k(t−t0)ae(t−t0)b
 MathType@MTEF@5@5@+=feaafiart1ev1aaatCvAUfKttLearuWrP9MDH5MBPbIqV92AaeXatLxBI9gBaebbnrfifHhDYfgasaacH8akY=wiFfYdH8Gipec8Eeeu0xXdbba9frFj0=OqFfea0dXdd9vqai=hGuQ8kuc9pgc9s8qqaq=dirpe0xb9q8qiLsFr0=vr0=vr0dc8meaabaqaciaacaGaaeqabaqabeGadaaakeaacqWGdbWqcqGH9aqpcqWGRbWAcqGGOaakcqWG0baDcqGHsislcqWG0baDdaWgaaWcbaGaeGimaadabeaakiabcMcaPmaaCaaaleqabaGaemyyaegaaOGaemyzau2aaWbaaSqabeaadaWcaaqaaiabcIcaOiabdsha0jabgkHiTiabdsha0naaBaaameaacqaIWaamaeqaaSGaeiykaKcabaGaemOyaigaaaaaaaa@41CB@ was suggested to obtain an aortic time-attenuation curve, which could be used to correct the recirculation effect. In the above formula, *C *and *t *represent density and time, respectively, whereas *a*, *b*, *t*_0 _are fitting parameters. It has been reported that the gamma variate model provides a poor fit to the time density curve when the contrast bolus is small and rapid [14]. In [15], an empirical formula C=Cpe−klog⁡e2(t−ta)(tp−ta)
 MathType@MTEF@5@5@+=feaafiart1ev1aaatCvAUfKttLearuWrP9MDH5MBPbIqV92AaeXatLxBI9gBaebbnrfifHhDYfgasaacH8akY=wiFfYdH8Gipec8Eeeu0xXdbba9frFj0=OqFfea0dXdd9vqai=hGuQ8kuc9pgc9s8qqaq=dirpe0xb9q8qiLsFr0=vr0=vr0dc8meaabaqaciaacaGaaeqabaqabeGadaaakeaacqWGdbWqcqGH9aqpcqWGdbWqdaWgaaWcbaGaemiCaahabeaakiabdwgaLnaaCaaaleqabaGaeyOeI0Iaem4AaSMagiiBaWMaei4Ba8Maei4zaC2aa0baaWqaaiabdwgaLbqaaiabikdaYaaalmaalaaabaGaeiikaGIaemiDaqNaeyOeI0IaemiDaq3aaSbaaWqaaiabdggaHbqabaWccqGGPaqkaeaacqGGOaakcqWG0baDdaWgaaadbaGaemiCaahabeaaliabgkHiTiabdsha0naaBaaameaacqWGHbqyaeqaaSGaeiykaKcaaaaaaaa@4B90@ was proposed, where *C*, *C*_*p *_and *t *represent concentration (density), maximum concentration, and time, respectively, whereas *k*, *t*_*a*_, *t*_*p *_are fitting parameters. This function was shown to be incapable of fitting the obtained 3D bolus profile by several trials. In [16], a lagged normal density function (i.e., the convolution of a Gaussian and an exponential function) was used to fit the bolus density at the femoral and dorsalis pedis arteries. Recently, [8] used an updated lagged normal density function by including the effects of the injection pattern. Using this approach, the bolus temporal profile was converted into a spatial profile, which was used to resist the noise and feature extraction errors in bolus tracking for X-Ray Peripheral Angiography. The fitting functions used in the above work focused on bolus attenuation at a specified location, but paid little attention to bolus dispersion in the artery. However, to design an adaptive controller for bolus chasing CTA, a whole picture of bolus density, including its attenuation along the distance and time axis, is crucial. To that end, we need to extend the above work to the 3D fitting.

In this work, we initially examined all three bolus fitting functions mentioned above, and found that the data were best-fitted by the lagged normal function. Therefore, the lagged normal density model

b=C×12πσe−(t−tc)22σ2⊗1τe−tτ     (1)
 MathType@MTEF@5@5@+=feaafiart1ev1aaatCvAUfKttLearuWrP9MDH5MBPbIqV92AaeXatLxBI9gBaebbnrfifHhDYfgasaacH8akY=wiFfYdH8Gipec8Eeeu0xXdbba9frFj0=OqFfea0dXdd9vqai=hGuQ8kuc9pgc9s8qqaq=dirpe0xb9q8qiLsFr0=vr0=vr0dc8meaabaqaciaacaGaaeqabaqabeGadaaakeaacqWGIbGycqGH9aqpcqWGdbWqcqGHxdaTdaWcaaqaaiabigdaXaqaamaakaaabaGaeGOmaidcciGae8hWdahaleqaaOGae83WdmhaaiabdwgaLnaaCaaaleqabaGaeyOeI0YaaSaaaeaacqGGOaakcqWG0baDcqGHsislcqWG0baDdaWgaaadbaGaem4yamgabeaaliabcMcaPmaaCaaameqabaGaeGOmaidaaaWcbaGaeGOmaiJae83Wdm3aaWbaaWqabeaacqaIYaGmaaaaaaaakiabgEPiepaalaaabaGaeGymaedabaGae8hXdqhaaiabdwgaLnaaCaaaleqabaGaeyOeI0YaaSaaaeaacqWG0baDaeaacqWFepaDaaaaaOGaaCzcaiaaxMaadaqadaqaaiabigdaXaGaayjkaiaawMcaaaaa@5486@

was adopted to fit our datasets. In (1), *t *is time, and *C*, *σ*, *t*_*c *_and *τ *are four parameters, which are functions of the position *z*. After integrating Equation (1), we obtained

b(t,z)=C2τ×e−1τ+(tcτ+σ22τ2)[erf(12σ(t−tc−σ2τ))−erf(12σ(−tc−σ2τ))],     (2)
 MathType@MTEF@5@5@+=feaafiart1ev1aaatCvAUfKttLearuWrP9MDH5MBPbIqV92AaeXatLxBI9gBaebbnrfifHhDYfgasaacH8akY=wiFfYdH8Gipec8Eeeu0xXdbba9frFj0=OqFfea0dXdd9vqai=hGuQ8kuc9pgc9s8qqaq=dirpe0xb9q8qiLsFr0=vr0=vr0dc8meaabaqaciaacaGaaeqabaqabeGadaaakeaacqWGIbGycqGGOaakcqWG0baDcqGGSaalcqWG6bGEcqGGPaqkcqGH9aqpdaWcaaqaaiabdoeadbqaaiabikdaYGGaciab=r8a0baacqGHxdaTcqWGLbqzdaahaaWcbeqaaiabgkHiTmaalaaabaGaeGymaedabaGae8hXdqhaaiabgUcaRmaabmaabaWaaSaaaeaacqWG0baDdaWgaaadbaGaem4yamgabeaaaSqaaiab=r8a0baacqGHRaWkdaWcaaqaaiab=n8aZnaaCaaameqabaGaeGOmaidaaaWcbaGaeGOmaiJae8hXdq3aaWbaaWqabeaacqaIYaGmaaaaaaWccaGLOaGaayzkaaaaaOWaamWaaeaacqWGLbqzcqWGYbGCcqWGMbGzdaqadaqaamaalaaabaGaeGymaedabaWaaOaaaeaacqaIYaGmaSqabaGccqWFdpWCaaWaaeWaaeaacqWG0baDcqGHsislcqWG0baDdaWgaaWcbaGaem4yamgabeaakiabgkHiTmaalaaabaGae83Wdm3aaWbaaSqabeaacqaIYaGmaaaakeaacqWFepaDaaaacaGLOaGaayzkaaaacaGLOaGaayzkaaGaeyOeI0IaemyzauMaemOCaiNaemOzay2aaeWaaeaadaWcaaqaaiabigdaXaqaamaakaaabaGaeGOmaidaleqaaOGae83WdmhaamaabmaabaGaeyOeI0IaemiDaq3aaSbaaSqaaiabdogaJbqabaGccqGHsisldaWcaaqaaiab=n8aZnaaCaaaleqabaGaeGOmaidaaaGcbaGae8hXdqhaaaGaayjkaiaawMcaaaGaayjkaiaawMcaaaGaay5waiaaw2faaiabcYcaSiaaxMaacaWLjaWaaeWaaeaacqaIYaGmaiaawIcacaGLPaaaaaa@808A@

where erf(s)=∫0se−t2dt
 MathType@MTEF@5@5@+=feaafiart1ev1aaatCvAUfKttLearuWrP9MDH5MBPbIqV92AaeXatLxBI9gBaebbnrfifHhDYfgasaacH8akY=wiFfYdH8Gipec8Eeeu0xXdbba9frFj0=OqFfea0dXdd9vqai=hGuQ8kuc9pgc9s8qqaq=dirpe0xb9q8qiLsFr0=vr0=vr0dc8meaabaqaciaacaGaaeqabaqabeGadaaakeaacqWGLbqzcqWGYbGCcqWGMbGzcqGGOaakcqWGZbWCcqGGPaqkcqGH9aqpdaWdXaqaaiabdwgaLnaaCaaaleqabaGaeyOeI0IaemiDaq3aaWbaaWqabeaacqaIYaGmaaaaaOGaemizaqMaemiDaqhaleaacqaIWaamaeaacqWGZbWCa0Gaey4kIipaaaa@4138@ is the error function. In Equation (1) and (2), *σ *and *τ *describe the bolus dispersion shape, *C *gives the magnitude of the bolus density, and *t*_*c*_, *σ *and *τ *approximately determine the time of peak bolus density at a fixed position. Our bolus fitting procedure was as follows:

1) The main part of the bolus was selected. Avoiding the lowest signal intensity portions of the bolus helps to make the fitting problem numerically well-conditioned.

2) At a given position *z*_*k*_, the parameters *σ*, *t*_*c*_, *C *and *τ *are fixed because they are functions of distance. The temporal curve at *z*_*k *_was found by adjusting *σ*, *t*_*c*_, *C *and *τ *to minimize the sum of the squares of the errors; that is, [C,σ,tc,τ]k=arg⁡min⁡[C,σ,tc,τ]{∑t[b(t,zk)−B(t,zk)]2}
 MathType@MTEF@5@5@+=feaafiart1ev1aaatCvAUfKttLearuWrP9MDH5MBPbIqV92AaeXatLxBI9gBaebbnrfifHhDYfgasaacH8akY=wiFfYdH8Gipec8Eeeu0xXdbba9frFj0=OqFfea0dXdd9vqai=hGuQ8kuc9pgc9s8qqaq=dirpe0xb9q8qiLsFr0=vr0=vr0dc8meaabaqaciaacaGaaeqabaqabeGadaaakeaadaWadaqaaiabdoeadjabcYcaSGGaciab=n8aZjabcYcaSiabdsha0naaBaaaleaacqWGJbWyaeqaaOGaeiilaWIae8hXdqhacaGLBbGaayzxaaWaaSbaaSqaaiabdUgaRbqabaGccqGH9aqpdaWfqaqaaiGbcggaHjabckhaYjabcEgaNjGbc2gaTjabcMgaPjabc6gaUbWcbaWaamWaaeaacqWGdbWqcqGGSaalcqWFdpWCcqGGSaalcqWG0baDdaWgaaadbaGaem4yamgabeaaliabcYcaSiab=r8a0bGaay5waiaaw2faaaqabaGcdaGadeqaamaaqafabaWaamWaaeaacqWGIbGycqGGOaakcqWG0baDcqGGSaalcqWG6bGEdaWgaaWcbaGaem4AaSgabeaakiabcMcaPiabgkHiTiabdkeacjabcIcaOiabdsha0jabcYcaSiabdQha6naaBaaaleaacqWGRbWAaeqaaOGaeiykaKcacaGLBbGaayzxaaWaaWbaaSqabeaacqaIYaGmaaaabaGaemiDaqhabeqdcqGHris5aaGccaGL7bGaayzFaaaaaa@6A39@ where *B*(*t*,*z*_*k*_) is actual density value at time *t *and position *z*_*k*_.

3) Steps 1 and 2 were repeated at every temporal position;

4) The parameters *σ*, *t*_*c*_, *C *and *τ *were expressed as a function of the position *z*.

**Figure 4:**
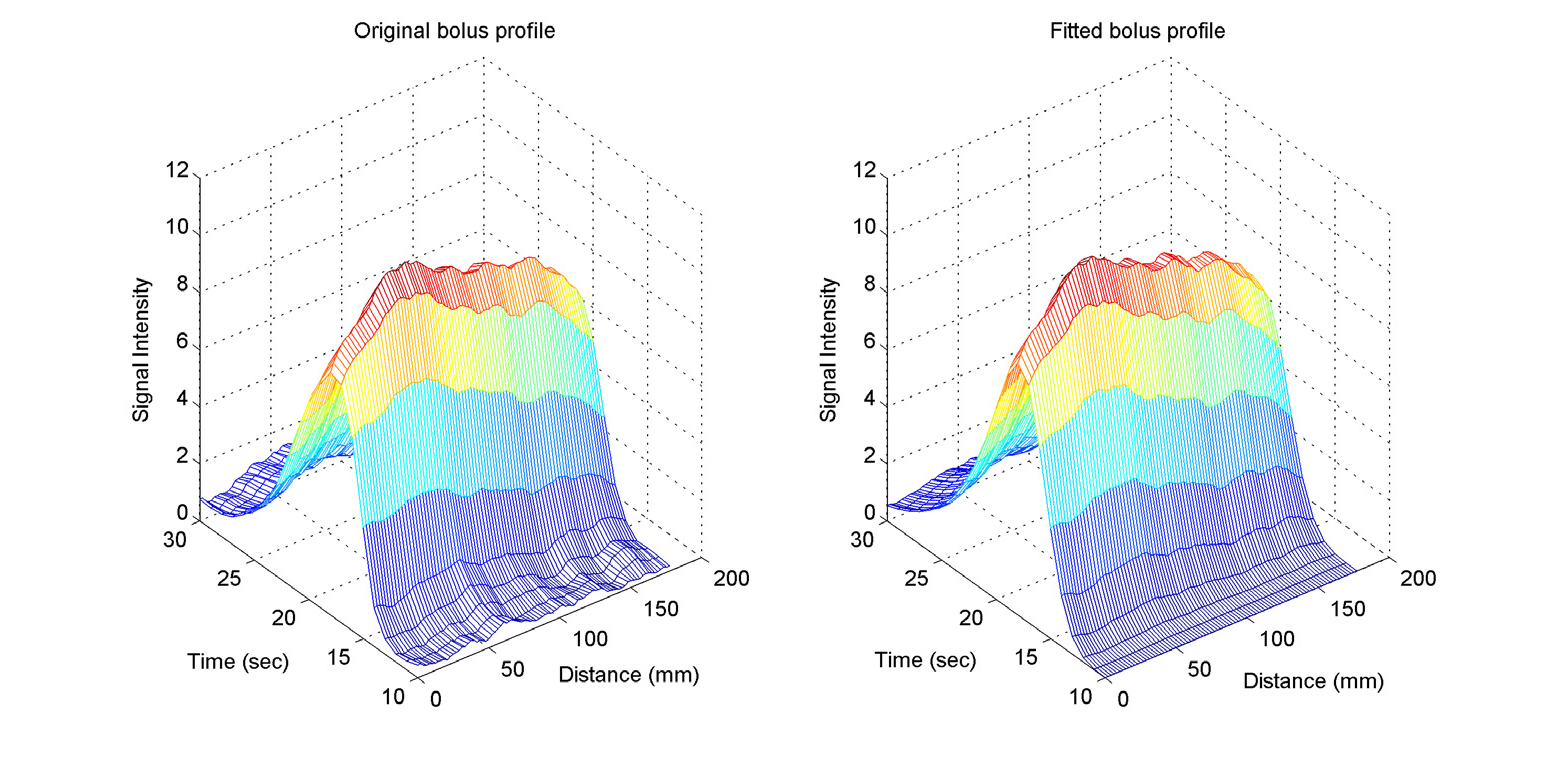
Left: Original bolus 3D profile extracted from MR data. Right: bolus 3D profile generated from the lagged normal density model using the best-fit parameters obtained from the data.

**Figure 5:**
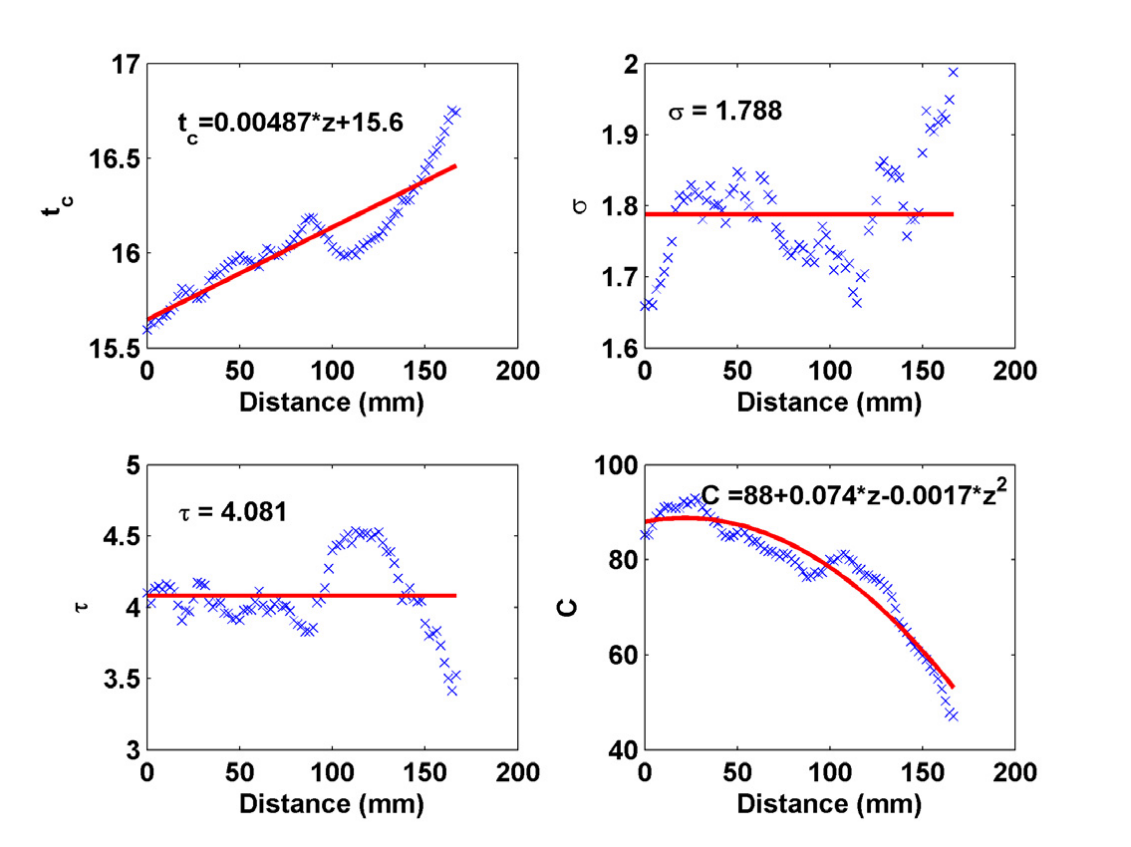
Fitting parameters *t*_*c *_*σ*, *τ *and *C *at different locations: × line denotes the fitting parameter values, and the solid line represents the approximate relationships of these parameters as functions of position.

A representative curve fit is shown in Figure 4. The 3D profile of the bolus fitted the original data well. Moreover, Equation (1) provided a good fit to all datasets, supporting its selection and use in the model. The next step was to find a relationship between the parameters (i.e., *σ*, *t*_*c*_, *C *and *τ*) and distance, and then construct a 3D model. A typical relationship between distance and the four parameters is shown in Figure 5.

**Figure 6:**
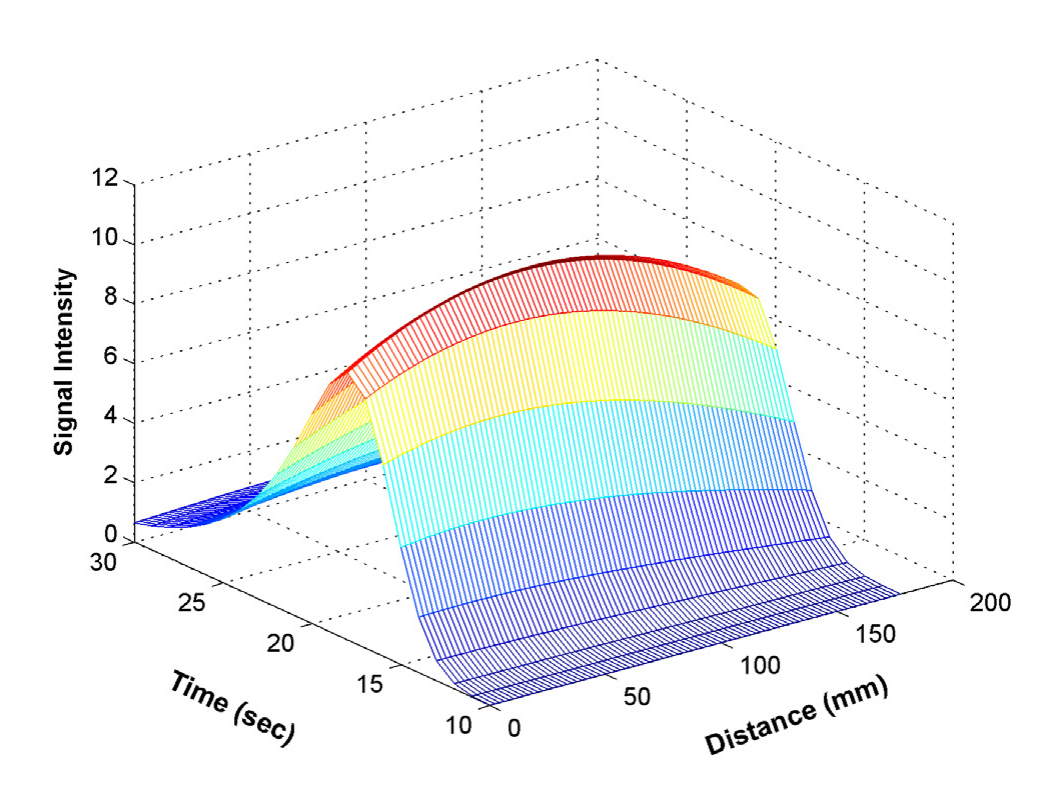
Bolus profile generated by Equation (2) using the approximate relationship between the fitting parameters and distance shown in Fig. 5.

In Figure 5, *σ *and *τ *varied modestly around some constant values, but there was no obvious functional relationship for them for any of the datasets. Therefore, we decided to treat them as constants by using their mean value. The other two fitting parameters, *t*_*c *_and *C*, were fit by using linear and quadratic functions, respectively. The reasons for selecting those functions were two-fold: first, the curves superficially resembled the corresponding functions; and secondly, *t*_*c*_, which represents the bolus peak time, would be expected to be a non-decreasing function. The density magnitude *C *would be expected to have a maximum and then decrease for increasing lengths. By using those parameters, we were able to generate a bolus propagation in the aorta from Equation (2). The resulting 3D profile is shown in Figure 6, where the unit of distance is the millimeter, and time ranges from 10 to 30 seconds. The parameters yielding the best-fit to the model for all thirty datasets are given in the Table 2.

**Table 1:** Summary of 30 MRA bolus timing datasets: selected features of interest and fitting errors.

*Source*	*Patient number*	*Patient Information*	*Bolus Velocity (cm/sec)*	*Travel Time (sec)*	*Travel Length (cm)*	*Relative Fitting error*
*UIHC 'GE' MR machine*	*1*	*M/50*	*9*	*10*	*25*	*0.122*
	*2*	*F/63*	*8*	*10*	*27*	*0.170*
	*3*	*M/15*	*9*	*10*	*28*	*0.056*
	*4*	*M/54*	*10*	*11*	*28*	*0.153*
	*5*	*F/21*	*13*	*7*	*23*	*0.039*
	*6*	*F/41*	*11*	*8*	*29*	*0.102*
	*7*	*M/71*	*6*	*7*	*16*	*0.046*
	*8*	*M/64*	*9*	*7*	*26*	*0.038*
	*9*	*F/49*	*5*	*10*	*14*	*0.051*
	*10*	*F/66*	*12*	*11*	*26*	*0.065*
	*11*	*M/51*	*7*	*9*	*19*	*0.107*
	*12*	*M/50*	*10*	*10*	*21*	*0.089*
	*13*	*M/23*	*6*	*8*	*23*	*0.093*
	*14*	*F/55*	*6*	*11*	*25*	*0.098*
	*15*	*M/45*	*6*	*8*	*23*	*0.129*
	*16*	*F/22*	*13*	*6*	*19*	*0.180*
	*17*	*F/60*	*8*	*7*	*21*	*0.064*
	*18*	*M/58*	*10*	*11*	*22*	*0.135*
	*19*	*F/25*	*10*	*11*	*24*	*0.087*
	*20*	*M/48*	*11*	*8*	*23*	*0.049*
	*21*	*M/26*	*6*	*12*	*26*	*0.063*
	*22*	*M/25*	*8*	*10*	*22*	*0.057*

*NU 'Siemens ' MR machine*	*23*	*M/34*	*13*	*8*	*23*	*0.005*
	*24*	*M/19*	*13*	*13*	*16*	*0.003*
	*25*	*F/40*	*8*	*8*	*21*	*0.008*
	*26*	*M/36*	*9*	*9*	*20*	*0.006*
	*27*	*M/57*	*10*	*12*	*21*	*0.003*
	*28*	*F/37*	*8*	*8*	*19*	*0.005*
	*29*	*M/34*	*9*	*10*	*11*	*0.024*
	*30*	*F/27*	*6*	*9*	*10*	*0.015*

Abbreviations: M = Male; F = Female

**Table 2:** Summary of bolus model fitting parameters.

	*patient number*	*t*_*c*_		*σ*	*τ*	*C*		
					
		*a*_0_	*a*_1_			*C*_0_	*C*_1_	*C*_2_
*UIHC 'GE' MR machine*	**1**	*15*	*0.0076*	*1.80*	*4.89*	*4.2*	*0.044*	*-1.2e-4*
	**2**	*17*	*0.017*	*7.75*	*5.25*	*4.4*	*-0.0032*	*-7e-5*
	**3**	*17*	***-0.0029***	*2.34*	*8.10*	*5.4*	*0.068*	*-5.2e-4*
	**4**	*13*	*0.019*	*2.04*	*5.30*	*2.7*	*0.014*	*-4e-5*
	**5**	*13*	*0.0063*	*2.06*	*5.17*	*4.7*	*0.026*	*-1.7e-4*
	**6**	*13*	*0.00047*	*1.67*	*6.37*	*5.1*	*-0.012*	*-4.3e-5*
	**7**	*22*	*0.044*	*2.81*	*19.70*	*5.5*	*0.25*	*-3e-3*
	**8**	*18*	*0.0089*	*2.06*	*6.79*	*5.8*	*-0.11*	*-2.4e-5*
	**9**	*14*	*0.029*	*2.63*	*7.72*	*2.6*	*0.056*	*-5.6e-4*
	**10**	*15*	*0.015*	*2.36*	*4.75*	*8.5*	*-0.052*	***3.5e-4***
	**11**	*16*	*0.0092*	*1.87*	*8.27*	*5.7*	*-0.021*	***4.4e-5***
	**12**	*15*	*0.0085*	*1.77*	*7.03*	*6.3*	*0.034*	*-4e-4*
	**13**	*16*	*0.001*	*2.05*	*4.81*	*5*	*0.016*	*-3.6e-5*
	**14**	*18*	*0.0082*	*2.48*	*18.20*	*16*	*-0.12*	***7.3e-4***
	**15**	*14*	*0.0096*	*2.02*	*4.98*	*6.2*	*0.012*	*-8.9e-5*
	**16**	*10*	*0.004*	*1.35*	*3.85*	*6.7*	*-0.051*	***1.8e-4***
	**17**	*14*	*0.0085*	*1.85*	*4.98*	*4.3*	*0.11*	*-6.5e-5*
	**18**	*12*	*0.0076*	*1.60*	*5.41*	*4.7*	*-0.021*	***1.4e-5***
	**19**	*15*	***-0.0034***	*1.77*	*5.42*	*7.2*	*-0.0014*	***4.7e-6***
	**20**	*13*	*0.0054*	*1.62*	*6.10*	*3.5*	*0.013*	*-9e-5*
	**21**	*15*	*0.013*	*2.25*	*12.13*	*8.1*	*-0.067*	***3.9e-4***
	**22**	*15*	*0.0062*	*2.24*	*9.16*	*14*	*-0.034*	*-1.3e-4*

*NU 'Siemens ' MR machine*	**23**	*16*	*0.0049*	*1.79*	*4.08*	*0.88*	*0.00074*	*-1.7e-5*
	**24**	*23*	*0.0089*	*2.99*	*7.42*	*1.4*	*-0.0087*	***7.4e-6***
	**25**	*24*	*0.0067*	*2.06*	*6.62*	*1.4*	*-0.0032*	*-1.4e-5*
	**26**	*25*	*0.0061*	*2.08*	*6.52*	*1.3*	*-0.0035*	*-1.5e-5*
	**27**	*28*	*0.0086*	*2.90*	*19.07*	*1.4*	*0.12*	*-1.3e-4*
	**28**	*15*	*0.0027*	*1.86*	*4.09*	*0.81*	*-0.0034*	***5.7e-6***
	**29**	*19*	*0.0078*	*2.62*	*3.48*	*1.5*	*0.036*	*-4.9e-4*
	**30**	*20*	*0.0043*	*2.63*	*4.25*	*0.66*	*0.019*	*-5e-4*

The fitting parameter *σ *and *τ *are represented by mean values, while *t*_*c *_and C are represented by linear function *t*_*c *_= *a*_0 _+ *a*_1_*z *and quadratic function *C *= *C*_0 _+ *C*_1_*z *+ *C*_2_*z*^2^.

## Results

The datasets from 30 patients were studied. Datasets were analyzed using the algorithm described above. Table 1 summarizes the bolus characteristics, where "*Bolus Velocity*", "*Travel Time*" and "*Travel Length*" were used to describe bolus characteristics, while *"Relative Fitting error" *shows the accuracy of the fitting. The "*Relative Fitting error*" is given by average fitting error square for each point divided by the maximum signal intensity of the dataset. From Table 1, we can see that most of the relative error is smaller than *0.1 *(22 out of 30), which shows the great fitting ability of the lagged normal density model. In Table 1, the fitting error for UIHC datasets is bigger than that for NU datasets, this may be due to the different MR machine technical parameters and it is beyond the scope of this paper. Because of the fast movement of contrast bolus in the aorta and poor temporal resolution of MRI, we did not extract bolus peak trajectory. Furthermore, in this paper, all of the datasets used a small amount of contrast material (2–3 cc) to determine the bolus arrival time in clinical MRA studies (see section 2.1). In clinical parlance, these datasets are referred to as test bolus or bolus timing runs. For these reasons, discussing bolus maximum velocity here may not be a good practical fit. In this study, "*Bolus Velocity*" for thirty datasets ranged from 5 to 13 cm/sec with average about 9 cm/sec. Compared to the reported blood average velocity in the aorta, which is about 27 cm/sec [17], the obtained velocity is relatively low. Considering the higher density of the contrast material and its dispersion, this is reasonable. Besides, the current clinical CT scanning protocols do not require CT table to be moved at a speed higher than 6 cm/sec.

**Figure 7:**
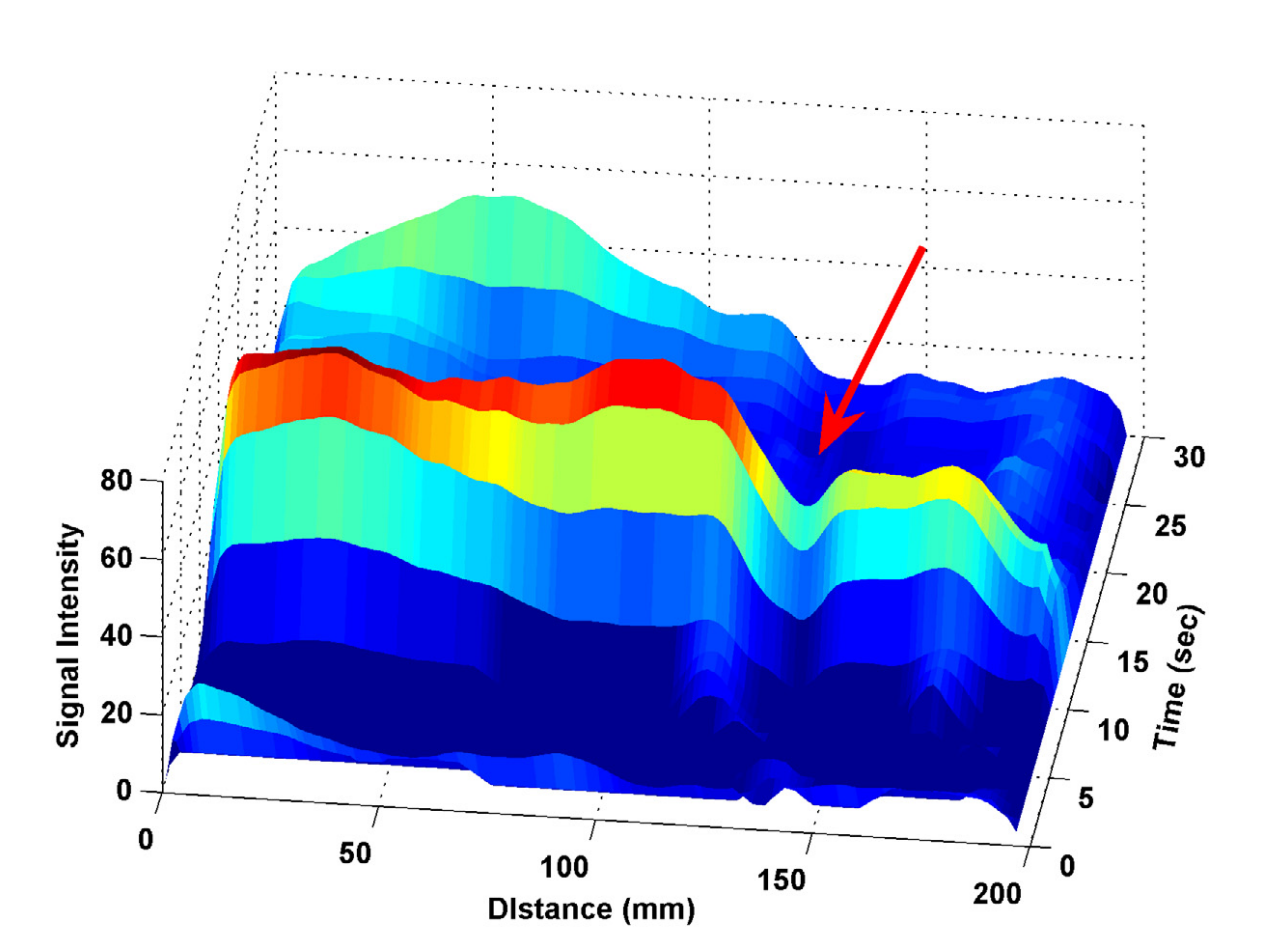
A 3D bolus profile with unexpected fitting parameters at one location. The bolus density is abnormally low at position around 145 mm all the time, which may be due to vessel curvature, narrowing, or problems.

The lagged normal density function fits the bolus datasets very well even though the original MRA images are noisy. In Table 2, the datasets from UIHC and NU had different magnitudes, which could reflect the different pulse sequence parameters used for the bolus timing runs (see section 2.1). For example, the value of the parameter *C *had a different magnitude for each of the two data sources. Table 2 summarized the bolus model parameters, where *C *= *C*_0 _+ *C*_1_*z *+ *C*_2_*z*^2 ^and *t*_*c *_= *a*_0 _+ *a*_1_*z *were functions of distance *z*, and *σ *and *τ *are constant (as explained in section 2.3). Intuitively, we expected the bolus peak to move forward as time progresses; we also expected the maximum density of the bolus to decrease as it travels distally along the artery. In the mathematical model of bolus propagation, these intuitive features were realized when *a*_1 _is positive and *C*_2 _is negative. However, the noise and other factors, such as the hemodynamic variability among patients and aortic locations, may confound the modeling and cause *a*_1 _to be negative or *C*_2 _positive (see bold and underlined cells in Table 2). An example of a positive *C*_2 _is shown in Figure 7, where a gap (indicated by the arrow) caused *C*_2 _to be positive. The gap could be due to vessel curvature, narrowing, or another problem. Although *t*_*c *_was fitted as a linear function, it varied over a very small range, which had an average about 2.2. This means that bolus peak remained in the aorta for about 2.2 seconds. Parameter *a*_0_, can be interpreted as the bolus arrival time in the proximal aorta. The shape of the bolus dispersion curve was determined by *σ *(normal distribution curve parameter) and *τ *(exponential curve parameter), whose average values were 2.31 and 7.33, respectively. Table 2 shows that bolus had very close *σ *value despite the variety of patients except patient 2. As for density level magnitude *C*, negative *C*_2 _means bolus density magnitude will decrease as distance increases after it reaches its maximum, which is the normal case during the bolus propagation because of dilution.

## Discussion

Analysis of MRA bolus timing data provides information about contrast bolus dynamics. The 3D bolus profile is a useful tool for simulating bolus-chasing CTA. The physicians always want the best diagnosis image, which requires the highest SNR (signal to noise ratio) at each vascular position for CT scanning. To that end, scanning each vascular position with highest density bolus inside is highly desired. However, during the CT scanning, it is very unlikely to know the maximum density time at each vascular position due to narrow imaging window of CT machine and complicated dynamics of contrast bolus. Therefore, an adaptive controller is urgently needed to predict the bolus maximum density time for the next scanned position. The controller requires the information of the bolus peak density in order to function correctly. On the other hand, the bolus fitting function (i.e., the propagation model) can provide the necessary information and be used to facilitate the controller design. For example, identification of *σ*, *t*_*c*_, *C *and *τ *in Equation (1) enables us to have an optimal trajectory of CT table movement. In fact, the more information about the propagating bolus, the better the controller design would be. Our ultimate goal is to design an adaptive controller, which is robust to track the bolus peak density of all patients with all diseases on CT machine. Currently, the CT machine operators moves CT table at a pre-set constant velocity, which does not consider the complicated bolus dynamics, the patient characteristics, and the disease effects. It is unlikely to have a good tracking result unless overdose is used. The adaptive controller will benefit both patient and physician in 1) better diagnosis CT image, 2) reduction of dose, 3) reduction of radiation exposure. In our latest work, we have designed an adaptive optimal controller, which has been proved to have an ability to track the bolus peak position very well and maximize the signal intensity at each scanned position.

Our analysis has several limitations related to the MR data. 1) Only a portion of the aorta was evaluated. For some CTA studies, the entire arterial tree from the chest to the toes will be scanned, but the bolus dynamics vary with arterial location. It is expected to have the MR sequence images of the entire arterial tree. 2) MR images demonstrate a complex and non-linear relationship between signal intensity and contrast bolus concentration. Thus, our analysis, which assumes a linear relationship, represents an approximation. 3) The temporal resolution of the MR data is less than that of CTA. The MR frame rate of one per second may be inadequate to capture some key features of the contrast bolus.

In conclusion, we have not only developed a tool for analyzing the characteristics of the MRA contrast bolus using a widely available software MATLAB and the Image Processing Toolbox, but found a mathematical model that fits the bolus well. Our results provided realistic simulations for bolus-chasing CTA and critical information for the design of an adaptive controller. Future work will focus on controller design and validation by simulation and experiment.

## Competing interests

The author(s) declare that they have no competing interests.
